# How psychological and descriptive narratives modulate the perception of facial emotional expressions: an event-related potentials (ERPs) study

**DOI:** 10.1007/s00221-025-07087-8

**Published:** 2025-04-30

**Authors:** Daniela Altavilla, Valentina Deriu, Alessandra Chiera, Stefania Crea, Ines Adornetti, Francesco Ferretti

**Affiliations:** Cosmic Lab, Department of Philosophy, Communication and Performing Arts, “Roma Tre” University, Via Ostiense, 234 00146 Rome, Italy

**Keywords:** Emotions, Narrative, Transportation, Social cognition, Facial emotional expressions, ERPs

## Abstract

The aim of the present study was to investigate whether stories with high and low narrative transport exert different effects on neural activation in response to facial emotional expressions. Thirty-one participants were randomly assigned to two groups based on the type of story they read: psychological narrative with high narrative transport (6 women and 10 men; age M = 34.38 ± 8.77); descriptive narrative with low narrative transport (9 women and 6 men; age M = 24.07 ± 7.38). The electroencephalographic activity of the participants in response to emotional facial expressions (joy, anger, fear, sadness) was recorded before (T0) and after (T1) the reading task. The findings indicated that the reading task modulated the early brain response (P1, N170) to emotional facial expressions, irrespective of the narrative type. However, only in the psychological narrative group was the amplitude of the P100 found to be positively associated with the extent to which an individual was transported into the narrative. In summary, the findings appear to indicate that an increased degree of transport into the narrative is associated with a greater internal simulation process of emotions and mental states. This, in turn, modulates the perception of the real social world after reading.

## Introduction

### ERPs and face processing

In the context of social interactions, faces serve as a rich source of information about others, including identity, mental states, and emotional states (e.g., Jack and Schyns 2015; Oruc et al. 2019a). Indeed, as highlighted by Oruc and colleagues (2019b), although the perception of a face is a remarkable computational achievement, it is recognized immediately and effortlessly by the human observer, this because an effective representation involves projecting a high-dimensional signal into a lower-dimensional space that retains the important characteristics of that signal for a particular task. In fact, a face representation can be conceptualized through semantic dimensions like facial attributes, or as a combination of fundamental functions that cover the so-called face-space.

From a neurophysiological perspective, event-related potentials (ERPs) studies have demonstrated a distinctive brain activation pattern associated with face processing. This is evidenced by a pronounced negative amplitude between 130 and 200 ms, commonly referred to as the N170 component, which is observed in response to facial compared to non-facial stimuli. This component is localized in occipital-temporal areas (e. (Bentin et al. 1996; Itier and Taylor 2004a; Roussel et al. 2004) and its source was identified in the fusiform gyrus and superior temporal sulcus (Itier and Taylor 2004b; Sadeh et al. 2010). Other ERP studies have also demonstrated that the N170 component is modulated by specific emotional facial expressions. Indeed, a meta-analysis conducted by Hinojosa and colleagues (2015) revealed that angry, fearful, and happy faces elicit greater N170 amplitudes compared to neutral facial expressions. Conversely, no significant results were observed for sad and disgusted faces. As posited by the authors, these results indicate that the processes underlying the N170 reflect a hierarchization of facial expressions likely not exclusively reliant on structural characteristics, but also on communicative and social factors. Indeed, unlike expressions of sadness or disgust, expressions of fear, anger or happiness require rapid socio-emotional reactions in the receiver, therefore, for their processing they would require rapid facial decoding mechanisms reflected by the N170 (Hinojosa et al. 2015). In addition to the N170, other early ERP components, such as the P100 and P200, and later components, such as the P300, appear to be modulated by emotion during face processing.

The P100 component is a pronounced positive amplitude that manifests between 80 and 120 milliseconds (ms) following the presentation of the stimulus. It is associated with the processing of perceptual information in visual brain regions, specifically the occipital lobes, and its amplitude is indicative of the recruitment of attentional control, selective attention, and the consumption of attentional resources (Allison et al. 1999; Dennis et al. 2007; Hillyard et al. 1995; 1998). The amplitude and latency of the P100 component are also influenced by emotional facial expressions. Indeed, several studies have found enhanced P100 amplitude in response to fearful, angry and happy faces in comparison to neutral faces (Batty and Taylor 2003; Kolassa et al. 2006; Moradi et al. 2017; Pourtois et al. 2004). Furthermore, a smaller amplitude was found in response to surprised faces compared to other emotional expressions (Meaux et al. 2014), and the latency was found to be faster for fear versus neutral faces (Williams et al. 2004). Additionally, several studies on patients diagnosed with schizophrenia (Caharel et al. 2007; Earls et al. 2016; Shah et al. 2018) have demonstrated a diminished amplitude and delayed latency in response to faces in patients when compared to healthy controls. Specifically, a reduced P100 amplitude was observed in response to sad, angry, and fearful facial expressions in patients compared to controls (Shah et al. 2018).

Nevertheless, a recent meta-analysis by Schindler and Bublatzky (2020) claims that the findings for the P100 cannot be interpreted in a univocal way as they are “highly diverse and either confirm (e.g., see Blechert et al. 2012; Foti et al. 2010; Muller-Bardorff et al. 2018) or reject emotional modulations (e.g., see Smith et al. 2013; Wieser et al. 2012)." (p. 364). In contrast, the data relating to two subsequent ERPs components, i.e. P200 and P300, appears to be considerably more consistent. P200 and P300 are typically present in the parietal and frontal brain regions and are associated with higher-order perceptual and attentional processing (Luck and Hillyard 1994; Moratti et al. 2011). In this context, Schindler and Bublatzky (2020) reported that greater amplitude of P200 and P300 is often found in response to angry and happy expressions (e.g., Carretié et al. 2013; Herbert et al. 2013; Karl et al. 2016; Liu et al. 2012; Santos et al. 2008; Smith et al. 2013; Syrjanen et al. 2018; Tortosa et al. 2013; Wu et al. 2019; Zhang et al. 2012), suggesting that these components are indeed modulated by emotional information.

### Face processing, social cognition, and narrative

In support of the fact that the face is a rich source of information about others and is central to social interactions, several ERP and neuroimaging studies have demonstrated a positive correlation between face processing and social cognition abilities. Social cognition is a multidimensional construct that enables the perception, interpretation, and effective response to social stimuli (Patin and Hurlemann 2015; Pinkham et al. 2014; Happé and Bird 2017; De Jaegher et al. 2010; Frith and Frith 2008). It encompasses both low-order and high-order processes, including affective and cognitive empathy. The former refers to “an emotional reaction of the observer when perceiving that another is experiencing or is about to experience an emotion” (Dvash and Shamay-Tsoory 2014, p. 283). Cognitive empathy is the ability “to understand the feelings of others without necessarily implying that the empathizer is in an affective state himself” (Walter 2012, p. 10). Social cognition also includes theory of mind, which is defined as the ability to attribute mental states and beliefs to oneself and others in order to interpret and predict behaviours (Westra and Carruthers 2018). In a study conducted by Petroni and colleagues (2011), it was demonstrated that an individual's ability to comprehend the mental states of others, as measured by the Reading the Mind in the Eyes Test (Baron-Cohen et al. 2001), was positively correlated with the amplitude of the N170 and the activation of the fusiform gyrus, i.e., brain source of the N170. In alignment with these findings, several studies (Choi and Watanuki 2014; Meaux et al. 2014) have also indicated that the early ERPs (P100, N170) are influenced by the emotional expressions conveyed by a face, and that emotional abilities are associated with the N170, P200, P300 and late positive potential.

In the context of studies that analyse social cognitive processes, there is an interesting line of research that aims to investigate these processes through narrative (e.g., Black et al. 2021; Buck et al. 2014; Eekhof et al. 2022; Kidd and Castano 2013; Mar 2018a, b). Indeed, some fundamental characteristics of stories make them particularly suitable for the study of social cognition (e.g., Herman 2013; Oatley 1995). These characteristics are essentially related to characters: understanding a story means (among other things) understanding the emotions, motivations and goals that underlie the characters’ actions in a story (Adornetti et al. 2023; Altavilla et al. 2024; Ferretti 2022). In other words, the understanding of a story is an exercise in social cognition. In this regard, a substantial body of behavioural research has demonstrated that reading fiction can facilitate the development of social cognitive abilities (e.g., Djikic et al. 2013; Kidd and Castano 2013; Koopman 2015; Mar and Oatley 2008, Mar et al. 2011; Paluck 2009). As posited by Mar and colleagues (2006, 2009, 2010), the act of reading a narrative prompts the reader to experience a range of emotions, which leave an impression on the individual. Furthermore, as Oatley (1999) posits, the act of engaging with fiction entails the simulation of authentic challenges and, consequently, engenders tangible ramifications for the reader. Frequently, when an individual engages with a fictional narrative, the act of identification with the characters and emotional involvement in the story leads to a state of sympathetic resonance with the characters, and potentially even the experience of the events of the story as if the reader were directly experiencing them (see also Bal and Veltkamp 2013). Indeed, it can be argued that fictional narratives are more effective than non-fiction because they engage readers in a simulation process, forming new mental associations through the characters’ social experiences (Foroni and Mayr 2005; Hakemulder 2000; Hodson et al. 2009; Oatley 1999).

Neurobiological studies appear to corroborate the findings of behavioural studies (Ferstl and von Cramon 2002; Kuperberg et al. 2006; Mason et al. 2008; Virtue et al. 2006). For example, Mason and Just (2006, 2009) discovered that particular brain networks are implicated in both theory of mind tasks and narrative comprehension. These include the dorsomedial prefrontal cortex (dmPFC), the right temporal parietal junction (TPj), and the posterior superior temporal sulcus (pSTS), which can be conceptualized as a protagonist (or agent) perspective interpreter network. In particular, the dorsomedial prefrontal cortex is activated throughout the processing of a narrative, acting as an executive processor (protagonist monitor). In contrast, the temporal-parietal network appears to function as a simulator, generating expectations to comprehend the protagonist's intentions (Mason and Just 2009).

It is crucial to acknowledge the observations made by Walkington and colleagues (2020), which highlight the pivotal role of identification and transportation in eliciting social processes during the act of reading a story. As mentioned earlier, identification refers to the process by which the reader becomes one with the character of the story “and reacts to his or her experiences as if they were happening to the viewer” (Sestir and Green 2010, p. 275). Transportation is defined as "the state of feeling cognitively, emotionally and imaginatively immersed in a narrative world" (*ibid*., p. 275). In fact, several studies have shown that individuals who are highly immersed in a story show greater empathic responses after a week than their less immersed counterparts (Bal and Veltkamp 2013). They are also more likely to engage in prosocial behaviours (Johnson 2012) and show a reduction in critical thinking (Van Laer et al. 2013).

### The present study

In light of the aforementioned background, the present study sought to investigate whether reading narratives that differ in their focus on the emotional and psychological dimensions of characters (and thus which might elicit varying levels of identification and transportation) modulates the neuronal response to facial expressions of diverse emotions. To the best of our knowledge, no event-related potentials (ERPs) studies have been conducted. The participants were divided into two groups with similar levels of empathy, and their electroencephalographic activity was recorded during a visual task involving images of facial emotional expressions of joy, fear, anger, and sadness. Each participant's neural response to facial expressions was recorded twice: before (T0) and after (T1) the reading of a narrative text, which was either psychological or descriptive in nature, according to their assigned group. Psychological narrative placed a strong emphasis on the characters' mental and emotional states, while descriptive narrative focused on describing the characters' actions and setting. The objective of this study was twofold: firstly, to examine the impact of psychological and descriptive narratives on the brain response to facial emotional stimuli; secondly, to determine the association between these brain responses and the extent to which individuals are transported into the narrative. The first hypothesis is that the psychological narrative group after the reading (T1) will exhibit a greater amplitude and an earlier latency of the ERPs components, particularly of the early components (P100 and N170), in response to the emotional facial expressions compared to T0 and compared to T1 of the descriptive narrative group. Moreover, it is predicted that this modulation will be found mainly in response to negative emotional facial expressions, given the presence of themes related to illness and mourning in the psychological narrative. The second hypothesis is that the transportation scores will be positively correlated with amplitude and negatively with latency of the earlier ERP components.

## Methods

### Participants

A total of forty-two participants were included in the study. The participants were divided into two groups: the first group, comprising twenty individuals, was assigned to the psychological narrative group (PG), while the second group, comprising twenty-two individuals, was assigned to the descriptive narrative group (DG). In order to be included in the study, participants had to be right-handed, free of any neurological and/or psychiatric diagnoses, and not be taking any medication or using any drugs. The present study was approved by the Ethics Committee of [Roma Tre University], and all participants provided informed consent prior to their involvement in the study.

### Stimuli

The stimuli were composed of 120 black-and-white facial emotional expressions, 30 for each of the four emotional conditions (joy, fear, anger, and sadness), and 40 black-and-white neutral object images, which served as filler items (e.g., umbrella, watch, scissors).

The facial emotional expressions were selected from the Extended Cohn-Kanade Dataset (CK+, Kanade et al. 2000; Lucey et al. 2010), which is a comprehensive dataset for action unit and emotion-specific expression. The facial expressions were extracted from videos in which the subjects' faces exhibited a shift from a neutral expression to a targeted peak expression. The videos were recorded at a rate of 30 frames per second with a resolution of 640×480 pixels. In the present study, the frame of the facial expressions exhibiting a peak expression of ≥ 60% was selected. The images utilized as fillers were obtained from the International Affective Picture System (IAPS; Lang et al. 1997).

### Procedure

**Fig. 1:**
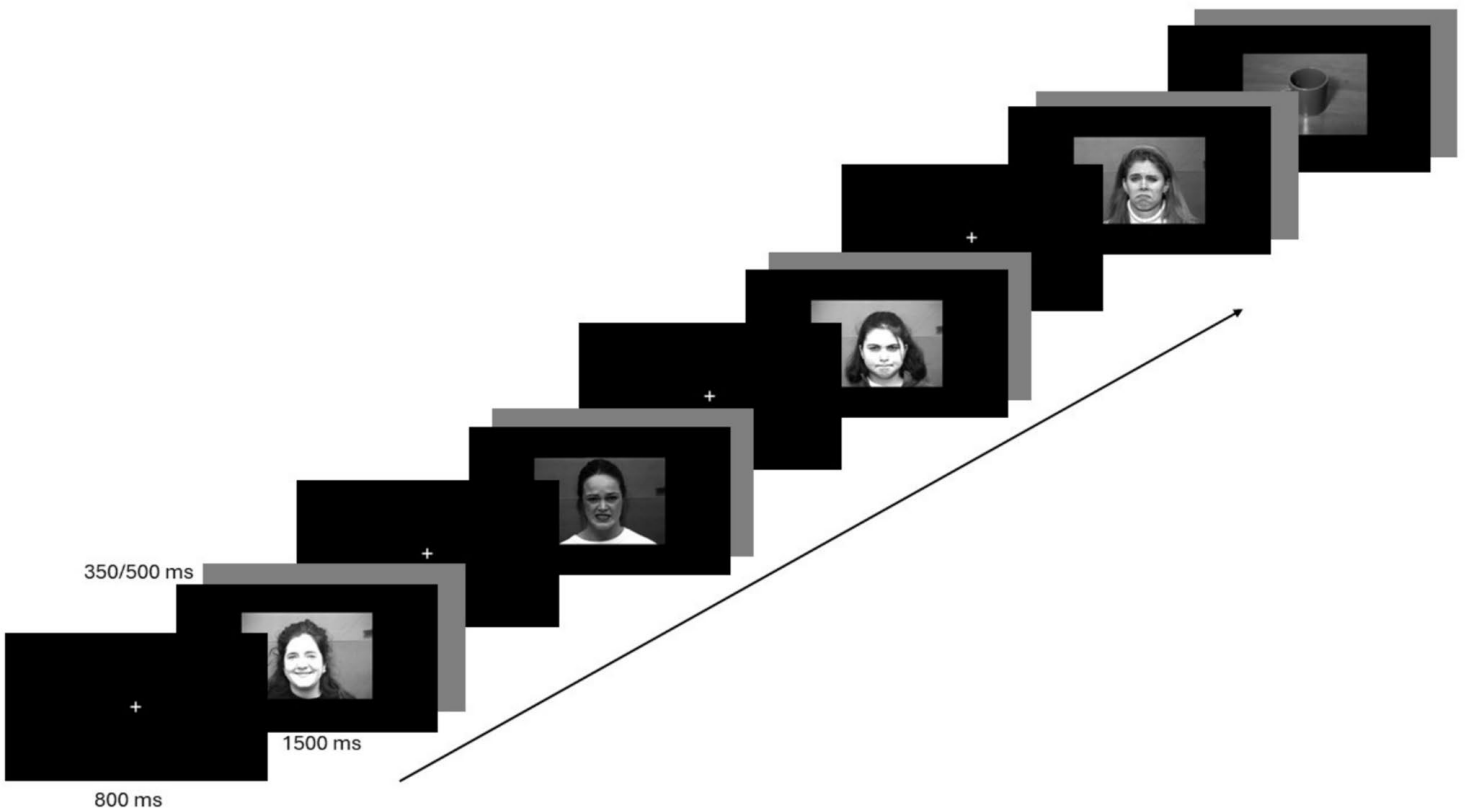
Experimental visual task

The participant was situated in front of a screen and outfitted with sensors on the head for the purpose of acquiring electroencephalographic data during a visual task. The visual experimental task was constructed using E-Prime software (version 2.0; Psychology Software Tools, Inc.). The visual task started with the follow instructions: “Please pay attention and watch the images that appear on the screen. When you are ready, press the space bar to begin.” As illustrated in Fig. 1, the trial began with the presentation of a fixation cross for a duration of 800 ms, after which the stimulus (joy, fear, anger, sadness, or filler) was displayed on the screen for a duration of 1500 ms. The trial was concluded with an inter-trial interval, which was randomly assigned to be either 350 or 500 ms. A total of 160 trials were presented in a randomized order.

Upon the conclusion of the initial presentation (T0), the instructions were displayed on the screen. *"Please read the text to your right in a safe and internal manner. You don't have a time limit. When you finish reading, press any key to restart the presentation."* The narrative text (psychological or descriptive) was placed on the desk at the participant's side. Right after reading the text, participants then repositioned themselves in front of the screen and pressed a button to restart the visual presentation of the stimuli (T1). The reading time of the participants of the psychological narrative group was M = 33’36” SD = 05’46’’, and of the descriptive narrative group was M = 35’41’’ SD = 07’28’’.

### Reading text

The aim of this study was to investigate the effect of narrative type on the neural response to facial emotional expressions. Behavioural studies (e.g., Bal and Veltkamp 2013) have shown that people are more transported into the story when it focuses on the mental and emotional states of the characters. As van Krieken et al. (2017, p. 4) observed, the significance of "privileged access to the perceptions, evaluations, and goals of a character" serves as a crucial mechanism through which readers are transported into the protagonist's world and able to identify with the characters. Based on this assumption, two different narratives were chosen: a psychological narrative, which emphasized the emotional and psychological dimension of the characters, and a descriptive narrative, which focused more on the narration of actions and the environmental context, or setting, of the story. Specifically, participants assigned to PG read the short story *A Small, Good Thing* from Raymond Carver's collection of short stories, *Cathedral* (1983, pp. 59–89, Italian translation 2014) (see “Appendix A”). This novel is about an eight-year-old boy named Scott who dies after being hit by a car on his way to school on his birthday. The selection of this text was driven by the fact that the entire narrative revolves around the emotions and mental states of the protagonists. This is exemplified by the boy's parents, who are portrayed as struggling with pain and feelings of uncertainty as they wait for their child to be hospitalized for three days. Given the prevalence of themes related to illness and mourning, as well as the emotions associated with them, such as fear, it was hypothesized that reading this type of narrative would facilitate transposition into the story and identification with the characters.

For the DG, participants read the short story “*Big two-hearted river*”, extracted from “*In our time*”, the inaugural American volume of short stories by Ernest Hemingway (1925; the Italian translation was extracted from the book “*Pietre, piume e insetti. L'arte di raccontare la natura*” edited by Sturani 2013, pp. 320–338). The story narrates the journey of a protagonist named Nick who returns to his father's town after the war and takes a path along the railroad tracks until he reaches a river (see “Appendix B”). In contrast to the psychological narrative, the focus of this novel is on the character's actions, such as fishing or eating trout, and his connection to nature. The environmental context is described in detail and with remarkable realism, while emotional or psychological dimensions are largely absent.

The two texts were converted to a uniform format. This involved transcribing them into a computer and reproducing the original organization in paragraphs, using the same font (Arial), size (15), and line spacing (1.5). The psychological and descriptive texts were 30 pages (9625 words) and 25 pages (7630 words), respectively. Participants read the printed version on white A4 paper, which did not indicate the title of the story. Therefore, they did not know which novel they were reading.

To ensure that the reading task was completed with the necessary degree of accuracy, a five-question comprehension test of the narrative was administered at the end of the experimental procedure. All participants achieved at least three correct answers and were included in the EEG data processing.

### Transportation and empathy assessment

To assess the extent to which participants were transported in the experimental reading narratives, Green and Brock's (2000, 2013) Transport Narrative Questionnaire was administered. The questionnaire consists of a 12-item scale that encompasses three dimensions of transport: cognition, emotion, and imagination. These three dimensions could be treated as subscales: cognitive (items 1, 3 and 4 from “Appendix C”), affective (items 5, 7, 11) and imagery (item 12). To the present study, only the total score of the transport scale was considered. Participants are asked to rate their experience of being immersed in a narrative on a 7-point Likert scale (1 = "not at all," 7 = "very much"), (see “Appendix C” for the full list of items).

To ensure that the results were not influenced by the different levels of empathy exhibited by the groups, empathy was assessed using the Interpersonal Reactivity Index (IRI) (Davis 1980), a 28-item self-report measure.

### Electroencephalographic data processing

Electroencephalographic data were recorded continuously at a sampling rate of 1000 Hz using Net Station software (version 5.3.0.1, Electrical Geodesic, Inc., Eugene, OR, USA) and a 64-hydrocel geodesic sensor net, with impedances kept below 50 kΩ and reference to the vertex (Cz).

EEG data were processed using Net Station software (*ibidem*). The digital 30Hz low-pass filter was applied offline. The EEG data of each participant were segmented into epochs from -100 ms to 600 ms after stimulus onset. A baseline correction was applied 100 ms before stimulus onset. Artifact detection was set to 200 μV for bad channels, 150 μV for eye blinks, and 100 μV for eye movements (Electrical Geodesic, Inc., Eugene, OR, USA; Altavilla et al. 2022; 2021; Cecchini et al. 2013; Chiera et al. 2022; McPartland et al. 2010; Picton et al. 2000). The segments with an eye blink, an eye movement or more than 30% bad channels were excluded.

The ERPs components extracted were: the peak amplitude and latency of the P100 (80–150 ms) on the occipital [left electrode: 35(O1); right electrode: 39(O2)] and temporal electrodes [left: 24(T7); 23(T9); right: 52(T8); 55(T10)], the peak amplitude and latency of the N170 (150–210 ms) on occipital, temporal and temporo-parietal electrodes [left: 25(TP7); 29(TP9); 27(P5); 30(P7); 32(P9); right: 48(TP8); 47(TP10); 45(P6); 44(P8); 43(P10)], and the mean amplitude of the P200 (210-350 ms) and P300 (350-450 ms) on temporo-parietal and fronto-central electrodes [left: 16(C1); 20(C3); 7(FC1); 15(FC3); 14(FC5); 9(F1); 12(F3); 13(F5); right: 51(C2); 50(C4); 54(FC2); 53(FC4); 57(FC6); 3(F2); 60(F4); 59(F6)].

### Statistical analysis

The repeated-measures ANOVAs 2 Group (psychological narrative *vs.* descriptive narrative) × 2 Time (pre- *vs*. post- reading)) × 4 Emotion (joy *vs.* fear *vs.* anger *vs.* sadness) × 2 Hemisphere (left *vs.* right) with Group as between factor and Time, Emotion, and Hemisphere as within factors were performed on the amplitude and latency of the P100 and N170, and on the amplitude of the P200 and P300 on each group of electrodes.

For each emotion, the repeated-measures ANOVAs 2 Group (psychological narrative *vs.* descriptive narrative) × 2 Time (pre- *vs.* post-reading) × 2 Hemisphere (left *vs.* right) with Group as between factor and Time and Hemisphere as within factors were performed on the amplitude and latency of the P100 and N170 and on the amplitude of the P200 and P300 on each group of electrodes. Fisher LSD post-hoc was applied.

Correlational analyses (*Pearson's r*) were performed between transport score and ERPs data at T1 for each emotion (joy, fear, anger, and sadness) in each group (psychological narrative and descriptive narrative). All statistical analyses were performed using Statistica v.8, StatSoft, Inc. 2007.

## Results

After cleaning the EEG data, only participants with at least 20% artifact-free trials in each condition at both recordings (T0 and T1) were included in the in statistical analyses: sixteen in the psychological narrative group (6 women and 10 men; age M = 34.38; SD = 8.77) and fifteen in the descriptive narrative group (9 women and 6 men; age M = 24.07; SD = 7.38). Means and standard deviation of the trials included in PG at T0 joy (24.63 ± 4.47), fear (24.94 ± 4.82), anger (23.88 ± 4.90), sadness (24.3 ± 5.08) and at T1 joy (22.88 ± 6.84), fear (22.3 ± 7.87), anger (22.75 ± 6.50), sadness (23.00 ± 5.85); in DG at T0 joy (22.47 ± 5,32), fear (23.47 ± 4.45), anger (23.67 ± 5.01), sadness (22.00 ± 5.36) and T1 joy (19.67 ± 7.28), fear (20.80 ± 7.34), anger (21.33 ± 5.73), sadness (20.80 ± 7.44).

The results of the independent t-test analysis indicated that there was no significant difference between the two groups on the total IRI empathy score [t_(29)_= − 1.25; *p* = 0.222]. However, a significant difference was observed between the two groups on the transportation score, with the participants of the psychological narrative group demonstrating a greater ability to transport themselves into the narrative compared to the participants of the descriptive narrative group [t_(29)_= 3.10; *p* = 0.004].

### ERPs amplitude

**Fig. 2:**
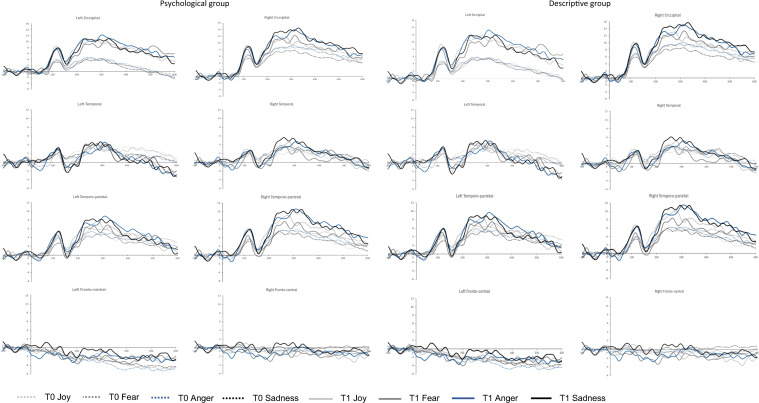
ERPs grand average of the emotions (joy, fear, anger and sadness) of both groups (psychological and descriptive) at T0 compared to T1 on left and right occipital, temporal, temporo-parietal and fronto-central electrodes.

As shown in Table 1, the 2 (Group) × 2 (Time) × 4 (Emotion) × 2 (Hemisphere) repeated-measures ANOVAs showed a main effect of Time in P100 on occipital [F(1,29) = 11.58; *p* = 0.002] and temporo-parietal [F(1,29) = 6.90; *p*= 0.014] electrodes, in N170 on temporal [F(1,29) = 13.08; *p*= 0.001] and temporo-parietal [F(1,29) = 12.43; *p* = 0.001] electrodes, with a lower amplitude at T0 compared to T1 (Fig. 2). The main effect of Hemisphere was found at P100, P200, and P300 in all electrode groups, with a lower amplitude in the left hemisphere compared to the right.

**Table 1:** ANOVAs Group [psychological narrative (PG) vs. descriptive narrative (DG)] × Time [pre-(T0) *vs.* post-(T1) reading] × Emotion [joy (J) *vs.* fear (F) *vs.* anger (A) *vs.* sadness (S)] × Hemisphere [left (L) *vs.* right (R)] on amplitude and *latency* in the P100 and N170, and on amplitude in the P200 and P300 on each left (L) and right (R) electrodes on which the components are extracted

Component	Electrodes Effects	Post-hoc
P100	*Occipital* Time F(1,29) = 11.58; *p* = 0.002 ηp^2^ = 0.29; G–G ε = 1	T0 < T1
	Hemisphere F(1,29) = 6.61; *p* = 0.016 ηp^2^ = 0.19; G–G ε = 1	L < R
	*Temporo-parietal* Time F(1,29) = 6.90; *p* = 0.014 ηp^2^ = 0.19; G–G ε = 1	T0 < T1
	Hemisphere F(1,29) = 5.97; *p* = 0.021 ηp^2^ = 0.17; G–G ε = 1	L < R
	Emotion × Hemisphere F(3,87) = 3.68; *p* = 0.015 ηp^2^ = 0.11; G–G ε = 0.77; G–G_(Adj)_ *p* = 0.025	**J (L)< J (R) *p* < 0.001**; **J (L)> A (L) *p* = 0.003** J (L)< S (R) *p* < 0.001; J (R)> F (L) *p*< 0.001 **J (R)> F (R) *p* < 0.001**; J (R)> A (L) *p* < 0.001 **J (R)> A (R) *p* < 0.001**; J (R)> S (L) *p* < 0.001 F (L)< S (R) *p*< 0.001; F (R)< A (L) *p*= 0.002 **F (R)< S (R) *p* < 0.001**; **A (L)< A (R) *p* =0.021** **A (L)< S (L) *p* = 0.008**; A (L)< S (R) *p* < 0.001 **A (R)< S (R) *p* < 0.001**; **S (L)< S (R) *p* < 0.001**
N170	*Temporal* Time F(1,29) = 13.08; *p* <0.001; ηp^2^ = 0.35; G–G ε = 1	T0 > T1
	*Temporal* *Time × Emotion × Hemisphere* *F(3,87) = 3.99; p* = 0.*010* ηp^2^ = 0.12;G–G ε = 0.89;G–G_(Adj)_ *p* = 0.013	***T0 A(L) > all conditions p <.01*** *T0 F(L) > T0 S(R) p = 0.035* *T0 F(L) > T1 F(R) p = 0.035* ***T0 F(R) > T0 S(R) p = 0.041*** ***T0 F(R) > T1 F(R) p = 0.042*** *T0 S(L) > T1 S(R) p = 0.045* *T0 S(L) > T1 F(R) p = 0.045*
	*Temporo-parietal* Time F(1,29) = 12.43; *p* = 0.001 ηp^2^ = 0.30; G–G ε = 1	T0 > T1
	Time × Hemisphere F(1,29) = 5.72; *p* = 0.023 ηp^2^ = 0.16; G–G ε = 1	**T0(L) > T0(R) *p* = 0.035** **T0(L) > T1(L) *p* < 0.001** T0(L) > T1(R) *p* < 0.001 T0(R) > T1(L) *p* < 0.001 **T0(R) > T1(R) *p* < 0.001**
P200	*Temporo-parietal* Hemisphere F(1,29) = 8.56; *p* = 0.007 ηp^2^ = 0.23; G–G ε = 1	L < R
	Time × Hemisphere F(1,29) = 4.56; *p* = 0.041 ηp^2^ = 0.14; G–G ε = 1	**T0(L) < T0 (R) *p* = 0.002** **T0(L) < T1(L) *p* < 0.001** T0(L) < T1(R) *p* < 0.001 **T0(R) < T1(R) *p* < 0.001** **T1(L) < T1(R) *p* < 0.001**
	*Fronto-central* Hemisphere F(1,29) = 10.87; *p* = 0.003 ηp^2^ = 0.27; G–G ε = 1	L < R
P300	*Temporo-parietal* Hemisphere F(1,29) = 10.41; *p* = 0.003 ηp^2^ = 0.26; G–G ε = 1	L < R
	*Fronto-central* Hemisphere F(1,29) = 13.78; *p*< 0.001 ηp^2^ = 0.32; G–G ε = 1	L < R

In bold the main post-hoc interaction effects discussed

The interaction effect of Time *per* Hemisphere was found in N170 [F(1,29) = 5.72; *p* =0.023] and P200 [F(1,29) = 4.56; *p* =0.041] on temporo-parietal electrodes where the right hemisphere at T1 presented a greater amplitude compared to left at T0 and at T1. An interaction effect Emotion *per* Hemisphere was found in P100 at temporo-parietal electrodes [F(3,87) = 3.68; *p* = 0.015], with joy eliciting a greater amplitude in the right hemisphere compared to anger and sadness, and sadness eliciting a greater amplitude in the right hemisphere compared to fear and anger (*p* < 0.001).

The 2 (Group) × 2 (Time) × 2 (Hemisphere) repeated-measures ANOVAs on the amplitude in response to each emotion showed the main effect of Time in P100 in response to joy [F(1,29) = 8.81; *p* = 0.006], fear [F(1,29) = 8.44; *p*= 0.007], anger [F(1,29) = 10.36; *p*= 0.003], and sadness [F(1,29) = 7.65; *p* = 0. 010] on occipital electrodes with lower amplitude at T0 compared to T1; in N170 in response to fear [F(1,29) = 6.67; *p* = 0.015] on temporo-parietal electrodes, in response to anger on temporal [F(1,29) = 6.84; *p* = 0.014] and temporo-parietal [F(1,29) = 9.94; *p* = 0.004] electrodes, and in response to sadness [F(1,29) = 4.94; *p* = 0.034] on temporal electrodes with less negative amplitude at T0 compared to T1. The main effect of hemisphere was found for the amplitude of almost all components and for almost all groups of electrodes, with the left hemisphere showing lower amplitude compared to the right hemisphere in response to all emotions. The interaction effect of Group *per* Time was found in P100 on occipital electrodes [F(1,29) = 4.59; *p* = 0.041] and in P300 on temporo-parietal electrodes [F(1,29) = 5.26; *p* = 0.031] in response to anger, with the descriptive narrative group presenting a greater amplitude at T1 compared to T0 and compared to the psychological narrative group at T1. The interaction effect of Group *per* Time *per* Hemisphere was found in N170 on temporal electrodes [F(1,29) = 4.28; *p* = 0.047] in response to joy, post hoc analyses showed that the descriptive narrative group presented a greater negative amplitude in the left hemisphere at T1 compared to T0. In Tables 1 and 2, all post-hoc results are shown.

**Table 2:** Significant effects of the ANOVAs Group [psychological narrative (PG) *vs.* descriptive narrative (DG)] × Time [pre-(T0) *vs.* post-(T1) reading] × Hemisphere [left (L) *vs.* right (R)] on each emotion (joy, fear, anger and sadness) on amplitude and *latency* in the P100 and N170, and on amplitude in the P200 and P300 on each left (L) and right (R) electrodes on which the components are extracted

Emotion	Component	Electrodes Effects	Post-hoc
Joy	P100	*Occipital* Time F(1,29) = 8.81; *p* = 0.006 ηp^2^ = 0.23; G–G ε = 1	T0 < T1
		Hemisphere F(1,29) = 6.38; *p* = 0.017 ηp^2^ = .18; G-G ε= 1	L < R
	N170	*Temporal* Group × Time × Hemisphere F(1,29) = 4.28; *p* = 0.047 ηp^2^ = 0.13; G–G ε = 1	**DG T0 L > DG T1 L *p* <0.001** DG T0 R > DG T1 L *p* = 0.004 DG T0 L > DG T1 R *p* = 0.013
	P200	*Temporo-parietal* Hemisphere F(1,29) = 7.08; *p* = 0.013 ηp^2^ = 0.20; G–G ε = 1	L< R
		*Fronto-central* Hemisphere F(1,29) = 6.25; *p* = 0.015 ηp^2^= 0.19; G–G ε = 1	L < R
	P300	*Temporo-parietal* Hemisphere F(1,29) = 7.99; *p* = 0.008 ηp^2^ = 0.22; G–G ε = 1	L < R
		*Fronto-central* Hemisphere F(1,29) = 7.21; *p* = 0.012 ηp^2^ = 0.20; G–G ε = 1	L < R
Fear	P100	*Occipital* Time F(1,29) = 8.44; *p* = 0.007 ηp^2^ = 0.23; G–G ε = 1	T0 < T1
		Hemisphere F(1,29) = 6.78; *p* = 0.014 ηp^2^ = 0.19; G–G ε = 1	L < R
		*Group × Hemisphere* *F(1,29)= 5.04; p* = *0.033* *ηp*^*2*^*= .15; G-G ε= 1*	***PG L < PG R p =0.041***
		*Group × Time × Hemisphere* *F(1,29) = 4.83; p* = *0.043* *ηp*^*2*^* = 0.13; G–G ε = 1*	*PG T0 L < PG T0 R p = 0.023*
	N170	*Temporo-parietal* Time F(1,29) = 6.67; *p* = 0.015 ηp^2^ = 0.19; G–G ε = 1	T0 > T1
		*Tempo-parietal* *Group × Time × Hemisphere* *F(1,29) = 7.16; p* = *0.012* *ηp*^*2*^* = 0.20; G–G ε = 1*	*PG T0 L > PG T0 R p = 0.014* *PG T0 R < PG T1 L p = 0.008* ***PG TO R < PG T1 R p = 0.013*** *DG T0 L < DG T0 R p = 0.020* ***DG T0 L < DG T1 L p = 0.018*** *DG T0 L < DG T1 R p = 0.049*
	P200	*Temporo-parietal* Hemisphere F(1,29) = 5.18; *p* = 0.030 ηp^2^ = 0.15; G–G ε = 1	L < R
		*Fronto-central* Hemisphere F(1,29) = 8.33; *p* = 0.007 ηp^2^ = 0.22; G–G ε = 1	L < R
	P300	*Temporo-parietal* Hemisphere F(1,29) = 5.22; *p* = 0.030 ηp^2^ = 0.15; G–G ε = 1	L < R
		*Fronto-central* Hemisphere F(1,29) = 13.46; *p* = 0.001 ηp^2^ = 0.32; G–G ε = 1	L < R
Anger	P100	*Occipital* Time F(1,29) = 10.36; *p* = 0.003 ηp^2^ = 0.26; G–G ε = 1	T0 < T1
		Group × Time F(1,29) = 4.59; *p* = 0.041 ηp^2^ = 0.14; G–G ε = 1	PG T0 < DG T1 *p* = 0.007 **DG T0 < DG T1 *p* < 0.001**
		*Temporal* *Time F(1,29) = 4.83; p* = *0.036* *ηp*^*2*^* = 0.14; G–G ε = 1*	*T0 > T1*
	N170	*Temporal* Time F(1,29) = 6.84; *p* = 0.014 ηp^2^ = 0.19; G–G ε = 1	T0 > T1
		*Temporo-parietal* Time F(1,29) = 9.94; *p* = 0.004 ηp^2^ = 0.26; G–G ε = 1	T0 > T1
		*Temporal* *Time × Hemisphere* *F(1,29) = 8.39; p* = *0.007* *ηp*^*2*^* = 0.22; G–G ε = 1*	*T0 L > T0 R p < 0.001* ***T0 L > T1 L p < 0.001*** *T0 L > T1 R p < 0.001*
	P200	*Temporo-parietal* Hemisphere F(1,29) = 7.47; *p* = 0.011 ηp^2^ = 0.20; G–G ε = 1	L < R
		*Fronto-central* Hemisphere F(1,29) = 11.56; *p* = 0.002 ηp^2^ = 0.29; G–G ε = 1	L < R
	P300	*Temporo-parietal* Hemisphere F(1,29) = 7.41; *p* = 0.011 ηp^2^ = 0.15; G–G ε = 1	L < R
		Gruppo × Time F(1,29) = 5.16; *p* = 0.031 ηp^2^ = 0.20; G–G ε= 1	**PG T1 < DG T1 *p* = 0.040**
		**Fronto-central** Hemisphere F(1,29) = 14.54; *p* < 0.001 ηp^2^ = 0.33; G–G ε = 1	L < R
Sadness	P100	*Occipital* Time F(1,29) = 7.65; *p* = 0.010 ηp^2^ = 0.21; G–G ε= 1	T0 < T1
		Hemisphere F(1,29) = 6.14; *p* = 0.019 ηp^2^ = 0.17; G–G ε = 1	L < R
	N170	*Temporal* Time F(1,29) = 4.94; *p* = 0.034 ηp^2^ = 0.15; G–G ε = 1	T0 > T1
	P200	*Temporo-parietal* Hemisphere F(1,29) = 7.65; *p* = 0.010 ηp^2^ = 0.21; G–G ε = 1	L < R
		*Fronto-central* Hemisphere F(1,29) = 10.357; *p* = 0.003 ηp^2^ = 0.27; G–G ε = 1	L < R
	P300	*Temporo-parietal* Hemisphere F(1,29) = 6.72; *p* = 0.015 ηp^2^ = 0.19; G–G ε= 1	L < R
		*Fronto-central* Hemisphere F(1,29) = 12.62; *p* = 0.001 ηp^2^ = 0.30; G–G ε = 1	L < R

In bold the main post-hoc interaction effects discussed

### ERPs latency

As shown in Table 1, the 2 (Group) × 2 (Time) × 4 (Emotion) × 2 (Hemisphere) repeated-measures ANOVAs showed an interaction effect of Time *per* Emotion *per* Hemisphere in N170 on temporal electrodes [F(3,87) = 3.99; *p* = 0.010], with anger showing a later latency at T0 compared to all conditions (*p* < 0.001), and fear showing a later latency at T0 compared to T1 in the right hemisphere.

The 2 (Group) × 2 (Time) × 2 (Hemisphere) repeated-measures ANOVAs on the latency in response to each emotion showed a main effect of time in P100 at temporal electrodes [F(1,29)= 4.83; *p* = 0.036] in response to anger, with shorter latency at T0 compared to T1.

An interaction effect of Group *per* Hemisphere was found in P100 at occipital electrodes [F(1,29)= 5.04; *p* = 0.033] in response to fear: the psychological narrative group showed a shorter latency in the left hemisphere compared to the right hemisphere. An interaction effect of Time per Hemisphere was found in N170 on temporal electrodes [F(1,29) = 8.39; *p* = 0.007] in response to anger: at T0, the left hemisphere showed a later latency compared to the right hemisphere, and at T1, it showed a later latency compared to T0. The interaction effect of Group per Time per Hemisphere was found at P100 on occipital electrodes [F(1,29) = 4.83; *p* = 0.043] and at N170 on temporo-parietal electrodes [F(1,29)= 7.16; *p* =0. 012] in response to fear: the results indicated that the psychological narrative group showed a later latency at T1 compared to T0 in the right hemisphere, while the descriptive narrative group showed a later latency at T1 compared to T0 in the left hemisphere. Tables 1 and 2 present the complete post hoc results.

### Correlation analyses between transportation score and ERPs data

As shown in Table 3, in the psychological narrative group, correlational analyses (*Pearson's r*) indicated a positive correlation between the transportation score and the amplitude of the P100 on the right occipital electrode in response to joy and on the right temporal electrodes in response to joy (r = 0.64; *p* = 0.008), fear (r = 0.51; *p* = 0.043), and sadness (r = 0.50; *p* = 0.048).

**Table 3:** Significant correlations (*Pearson’s r*) between transportation score and amplitude and *latency* of ERP components (P100, N170, P200, P300) at T1 on each left (L) and right (R) electrodes on which the components are extracted

	Group	Emotion	Component			
			P100	N170	P200	P300
Transportation score	Psychological narrative	Joy	R Temporal r = 0.64; *p* = 0.008	ns	ns	ns
		Fear	R Occipital r = 0.51; *p* = 0.043 R Temporal r= .59; *p* = 0.016	ns	ns	ns
		Anger	ns	ns	ns	ns
		Sadness	R Temporal r = 0.50; *p* = 0.048	ns	ns	ns
	Descriptive narrative	Joy	ns	*R Temporal* *r =0.54; p = 0.037*	ns	ns
		Fear	ns	ns	ns	ns
		Anger	ns	ns	ns	ns
		Sadness	ns	ns	ns	ns

In the descriptive narrative group, there was a positive correlation between transportation score and N170 latency in response to joy on the right temporal electrodes (r = 0.54; *p* = 0.037).

## Discussion

The face is one of the most informative and complex vehicles of social interaction. Several ERPs and neuroimaging studies have shown that the processing of faces and facial emotional expressions is modulated by social cognitive abilities. In recent years, a significant body of research has demonstrated the efficacy of narrative as a tool for investigating social cognition and its impact on social processes (Djikic et al. 2013; Kidd and Castano 2013; Koopman 2015; Mar and Oatley 2008, Mar et al. 2011; Paluck 2009). These studies have consistently shown that reading narrative influences social cognition, particularly when individuals report high levels of engagement with the story. To date, however, no neurophysiological studies appear to have examined whether reading a narrative that focuses more on the mental and emotional states of the character modulates neural activation in response to facial emotional expressions differently from reading a descriptive and more neutral narrative. Furthermore, it is unclear whether this activation correlates with the degree to which an individual is transported into the story. To address this question, the present study recorded the electrical brain activity of participants in two groups, the psychological narrative group and the descriptive narrative group, in response to facial emotional expressions (joy, anger, fear, and sadness) before and after a reading task.

The primary results showed a main effect of Time on the P100 at occipital and temporo-parietal electrodes, and the N170 at temporal and temporo-parietal electrodes, with greater amplitude observed at T1 compared to T0. The ANOVAs on each emotion also showed a main effect of Time in the P100 in response to joy, anger, and sadness on occipital electrodes, with greater amplitude at T1 compared to T0, and in the N170 in response to fear, anger, and sadness on temporo-parietal electrodes, and anger on temporal electrodes, with greater negative amplitude at T1 compared to T0.

Previous studies have shown a correlation between greater electrophysiological activation, particularly in the temporal N170, and improved social cognition (Choi and Watanuki 2014; Meaux et al. 2014; Petroni et al. 2011). Although emotional recognition was not assessed in the present study, the results seem to indicate that reading narratives, whether psychological or descriptive, increases attention to socioemotional stimuli.

It is crucial to highlight the early onset of this process, as evidenced by the observed effects on the amplitude of the early ERP components (P100 and N170), which are associated with low-order perceptual processes. This perceptual modulation can be explained by the fact that, as claimed by several authors (Mar and Oatley 2008; Mar et al. 2009; 2010; 2018; Mason and Just 2009; Oatley 1999), narration seems to provide an opportunity to simulate social scenarios, assume different perspectives, understand and anticipate the mental states and goals of the characters. This internal simulation "training" elicits greater socio-emotional perception of the real world. Consistent with these assumptions, the finding is also present in the group that reads the descriptive narrative probably because even in the case in which only the character's actions are described, and not his emotions and mental states, simulation and transport processes are activated to understand his intentions and goals.

Our results also revealed a significant Time *per* Emotion *per* Hemisphere interaction effect in latency. A post-hoc analysis indicated a shorter latency of the N170 in response to fear on the right temporal electrodes and in response to anger on the left ones after reading (T1) both psychological and descriptive narratives compared to T0. This result seems to confirm the previous interpretation and also suggests a faster perception of fear and anger facial expressions after reading the narrative, with a hemispheric lateralization related to the type of emotion. In line with the evolutionary approach to affective neuroscience research (e.g., Montag and Panksepp 2016; 2017; Panksepp 1998), fear and anger are considered negative basic emotions that are beneficial for individual survival, as they trigger the activation of a "fight-flight-freeze" system in dangerous situations. Greater and faster perceptual sensitivity to these emotions in a social context could facilitate more effective emotional regulation by enhancing emotional responsiveness and synchronization with others during social interaction.

Consistent with the latter point, the interaction effect of Group *per* Time *per* Hemisphere showed a longer latency of the N170 at temporo-parietal electrodes to fear following reading. This was observed specifically in the right hemisphere in the psychological narrative group and in the left hemisphere in the descriptive narrative group. This prolonged latency may indicate that after a rapid initial processing of emotion, more extended temporal processing in the temporo-parietal regions is required for the implementation of top-down emotional regulation.

By returning to the hemispheric lateralization, the greater involvement of the left hemisphere in response to anger and the right hemisphere in response to fear can be explained by reference to the approach/withdrawal motivational dimension of emotions, in line with several previous studies that have used visual and auditory emotional stimuli (Carver and Harmon-Jones 2009; Demaree et al. 2005; Gadea et al. 2011; Palomero-Gallagher and Amunts 2022). Consistent with the results of the present study, these investigations have shown that emotions that direct the individual toward the stimulus, such as anger and joy, are associated with the left hemisphere, whereas emotions that motivate withdrawal from the stimulus, such as fear and sadness, are associated with the right hemisphere.

Furthermore, it is important to highlight that, unlike several studies in which participants explicitly pay attention to or label the presented emotions into categories as well as some studies that have directly incorporated different tasks to assess to what extent task demands can modulate ERPs based on emotional faces (e.g., Wronka and Walentowska 2011; Rellecke et al. 2012; Itier and Neath-Tavares 2017; see Schindler et al. review 2023), in the present study the different neural response to different emotions was detected during an implicit task in which no explicit action of the participant was required suggesting bottom-up processes of emotional discrimination. In future research it would be interesting to include an explicit recognition task to also evaluate high-order processes.

Another important result was the interaction effect of Group *per* Time found in P100 on occipital electrodes and in P300 on temporo-parietal electrodes in response to anger with the descriptive narrative group presenting a greater amplitude at T1 compared to T0 and compared to psychological narrative group at T1, and the interaction effect of Group *per* Time *per* Hemisphere found in N170 on temporal electrode in response to joy with the descriptive narrative group presenting a greater negative amplitude in the left hemisphere at T1 compared to T0. The greater post-reading amplitudes observed in the descriptive narrative group can be attributed to the type of emotion evoked by the narrative. This narrative is characterized by an extremely long, emphatic, and meticulous description of details, including both the actions of the character and the description of the setting. The passage about the trout in the river (see “Appendix B”) is a prime example. Such a narrative may have elicited feelings of nervousness and boredom, which in turn may have more strongly modulated the response to emotions such as anger.

As predicted by the experimental manipulation, the psychological narrative group was found to have a higher transport score than the descriptive group. The psychological narrative may have evoked strong feelings of fear and distress, given the theme of illness and grief. Correlation analysis showed a positive correlation between transport score and P100 amplitude in response to joy, fear, and sadness only in this group after reading. The strong correlation also with joy shows that the more one immerses oneself in a stressful text, the greater the neural response to a positive emotion seems to be, suggesting the detection of an emotional incongruence with respect to the themes of illness and mourning present in the psychological narrative.

These results are of great importance for the purposes of the present study, as it supports the hypothesis that transportation into the story mediates the early brain perceptive response to socio-emotional stimuli, as shown by several behavioural studies (Bal and Veltkamp 2013; Johnson 2012; Sestir and Green 2010; Walkington et al. 2020). The greater the transport into the narrative, the greater the internal simulation process of emotions and mental states, which will modulate the perception of the real social world after reading. On the contrary, in the descriptive group, the transportation score correlates only with the latency of the N170 in response to joy, so immersion in an emotionless narrative seems to delay the processing of a positive emotion.

Finally, the interaction effect of Time *per* Hemisphere found at N170 and P200 on temporo-parietal electrodes showed a greater amplitude of the right hemisphere at T1 compared to the left at T0 and at T1. The greater involvement of the right hemisphere after reading could support the interpretation of greater attention to socioemotional stimuli. Indeed, several electrophysiological and neuroimaging studies show that the brain correlate of emotional processes and social skills, such as empathy and theory of mind, predominantly involves the right hemisphere (Adolphs 2002; Adolphs et al. 2000; Damasio 1994; Schore 2005). In this regard, the results of a recent ERP study (Altavilla et al. 2024) investigating how first- and third-person narrative modulates social cognitive ability also report a greater involvement of the right hemisphere. In this study, neural responses to eye expressions were recorded during an experimental explicit recognition task before and after reading the narrative. The results showed a smaller N220-400 on right fronto-central electrodes in response to eye expressions in both groups, and a larger N100 on left fronto-central electrodes and a larger P220-400 on right temporo-parietal electrodes in response to eye expressions only in the group reading the third-person story. No significant differences were found in the behavioural accuracy data after the reading task, probably due to the small sample size.

Overall, given the findings, it is plausible to hypothesize that the results of the present study may also be consistent with previous behavioural studies that have demonstrated improvements in social cognition following narrative reading (Djikic et al. 2013; Kidd and Castano 2013; Koopman 2015; Mar and Oatley 2008; Mar et al. 2011; Paluck 2009). However, further research using a behavioural emotion recognition task and a larger sample size, as also suggested by Altavilla and colleagues (2024), is needed to substantiate this claim.

In fact, these last points can be seen as two of the limitations of the present study: missing behavioural data and the small sample size. The small sample size is due to the double electroencephalographic recording, which led to the exclusion of eleven participants due to the presence of excessive artefacts. Although other ERP studies have reported similar sample sizes (e.g., Altavilla et al. 2022; 2024; Massaro et al. 2018; Ernst et al. 2013), it would be important to increase the sample size in future research to reduce the presence of artefacts due to double recording. One possibility would be to project the text onto the screen instead of providing it in paper form. Although this would reduce the naturalness and spontaneity of the reading, it might increase the likelihood of having a lower number of artefacts. It is also important to emphasise that, given the exploratory nature of the study, the data were not corrected for multiple comparisons, so further studies with even larger samples of homogeneous age are needed to generalise the data of the present study. Finally, in future research, a larger sample size with men and women homogeneously distributed across groups would also allow for gender-based analyses.

In conclusion, the present study suggests that reading narratives, whether psychological or descriptive, modulates the early brain response to socioemotional stimuli, i.e., facial emotional expressions. Moreover, this modulation is positively related to the degree to which an individual allows himself or herself to be transported into the story. Regarding this last point, it would be beneficial to conduct further research in the future to investigate the individual differences that might influence the ability to be transported into a story, including psychological variables, emotional regulation abilities, and attachment styles.

## Data Availability

No datasets were generated or analysed during the current study.

## Appendix A: An excerpt of the psychological narrative text

*[…] "Why won't he wake up?" Ann said. "Howard? I want some answers from these people." Howard didn't say anything. He sat down again in the chair and crossed one leg over the other. He rubbed his face. He looked at his son and then he settled back in the chair, closed his eyes, and went to sleep. Ann walked to the window and looked out at the parking lot. It was night, and cars were driving into and out of the parking lot with their lights on. She stood at the window with her hands gripping the sill and knew in her heart that they were into something now, something hard. She was afraid, and her teeth began to chatter until she tightened her jaws. She saw a big car stop in front of the hospital and someone, a woman in a long coat, get into the car. She wished she were that woman and somebody, anybody, was driving her away from here to somewhere else, a place where she would find Scotty waiting for her when she stepped out of the car, ready to say Mom and let her gather him in her arms.* *In a little while, Howard woke up. He looked at the boy again. Then he got up from the chair, stretched, and went over to stand beside her at the window. They both stared out at the parking lot. They didn't say anything. But they seemed to feel each other's insides now, as though the worry had made them transparent in a perfectly natural way.*

## Appendix B: An excerpt of the descriptive narrative text

*[…] The river was there. It swirled against the log spiles of the bridge. Nick looked down into the clear, brown water, coloured from the pebbly bottom, and he watched the trout keeping themselves steady in the current with fin wavering As he watched them they changed their positions by quick angles, only to hold steady in the fast water again. Nick watched them a long time! He watched them holding themselves with their noses into the current, many trout in deep, fast-moving water, slightly distorted as he watched far down through the glassy convex surface of the pool, its surface pushing and swelling smooth against the resistance of the log-driven piles of the bridge. At the bottom of the pool were the big trout. Nick didn't see them at first. Then he saw them at the bottom of the pool, big trout looking to hold themselves on the gravel bottom in a varying mist of gravel and sand, raised in spurts by the current! […]* *He saw the trout in the water jerking with his head and body against the shifting tangent of the line in the stream. Nick took the line in his left hand and pulled the trout, thumping tiredly against the current, to the surface. flis back was mottled the clear, water-over-gravel colour, his side flashing in the sun. The rod under his right arm, Nick stooped, dipping his right hand into the current. He held the trout, never still, with his moist right hand, while he unhooked the barb from his mouth, then dropped him back into the stream.*

## Appendix C: Questionnaire on narrative transportation (translated in Italian from Green and Brock 2000, 2013)

Circle the number (from1-Not at all to 7-Very much) under each question that best represents your opinion about the narrative you just read.

1. While I was reading the narrative, I could easily picture the events in it taking place.
2. While I was reading the narrative, activity going on in the room around me was on my mind.
3. I could picture myself in the scene of the events described in the narrative.
4. I was mentally involved in the narrative while reading it.
5. After the narrative ended, I found it easy to put it out of my mind.
6. I wanted to learn how the narrative ended.
7. The narrative affected me emotionally.
8. I found myself thinking of ways the narrative could have turned out differently.
9. I found my mind wandering while reading the narrative.
10. The events in the narrative are relevant to my everyday life.
11. The events in the narrative have changed my life.
12. I had a vivid mental image of [character name].

Notes: Items 2, 5, and 9 are reverse-scored.

Item 12 can be repeated for the number of main characters in the story, substituting a different

character name for each item.

## References

[CR1] Adolphs R (2002) Neural systems for recognizing emotion. Curr Opin Neurol 12(2):169–177. 10.1016/S0959-4388(02)00301-X 10.1016/s0959-4388(02)00301-x 12015233

[CR2] Adolphs R, Damasio H, Tranel D, Cooper G, Damasio AR (2000) A role for somatosensory cortices in the visual recognition of emotion as revealed by three-dimensional lesion mapping. J Neurosci 20(7):2683–2690. 10.1523/JNEUROSCI.20-07-02683.2000 10729349 10.1523/JNEUROSCI.20-07-02683.2000 PMC6772225

[CR3] Adornetti I, Chiera A, Deriu V, Altavilla D, Ferretti F (2023) Comprehending stories in pantomime. A pilot study with typically developing children and its implications for the narrative origin of language. Lang Commun 93:155–171

[CR4] Allison T, Puce A, Spencer DD, McCarthy G (1999) Electrophysiological studies of human face perception. I: Potentials generated in occipitotemporal cortex by face and non-face stimuli. Cereb Cortex. 9(5):415–30. 10.1093/cercor/9.5.415. (**PMID: 10450888**) 10450888 10.1093/cercor/9.5.415

[CR5] Altavilla D, Ciacchella C, Pellicano GR, Cecchini M, Tambelli R, Kalsi N, Lai C (2021) Neural correlates of sex-related differences in attachment dimensions. Affect Behav Neurosci 21:191–211. 10.3758/s13415-020-00859-5 10.3758/s13415-020-00859-5 PMC7994245 33560494

[CR6] Altavilla D, Adornetti I, Chiera A, Deriu V, Acciai A, Ferretti F (2022) Introspective self-narrative modulates the neuronal response during the emphatic process: an event-related potentials (ERPs) study. Exp Brain Res 240(10):2725–2738. 10.1007/s00221-022-06441-4 36066588 10.1007/s00221-022-06441-4

[CR7] Altavilla D, Adornetti I, Deriu V, Chiera A, Ferretti F (2024) Exploring how first-and third-person narrative modulates neural activation during a social cognition task. An event-related potentials (ERPs) study. Soc Neurosci 19(5–6):307–325 39694051 10.1080/17470919.2024.2441524

[CR8] Bal MP, Veltkamp M (2013) How does fiction reading influence empathy? An experimental investigation on the role of emotional transportation. PloS One 8:1–12. 10.1371/journal.pone.0055341 10.1371/journal.pone.0055341 PMC3559433 23383160

[CR9] Baron-Cohen S, Wheelwright S, Hill J, Raste Y, Plumb I (2001) The “Reading the Mind in the Eyes” Test revised version: a study with normal adults, and adults with Asperger syndrome or high functioning autism. J Child Psychol Psychiatry 42:241–251 11280420

[CR10] Batty M, Taylor MJ (2003) Early processing of the six basic facial emotional expressions. Brain Res Cogn Brain Res 17:613–620 14561449 10.1016/s0926-6410(03)00174-5

[CR11] Bentin S, Allison T, Puce A, Perez E, McCarthy G (1996) Electrophysiologicalstudies of face perception in humans. J Cogn Neurosci 8:551–565. 10.1162/jocn.1996.8.6.551 20740065 10.1162/jocn.1996.8.6.551 PMC2927138

[CR12] Black JE, Barnes JL, Oatley K, Tamir DI, Dodell-Feder D, Richter T, Mar RA (2021). Stories and their role in social cognition. Handbook of empirical literary studies 229–250

[CR13] Blechert J, Sheppes G, Di Tella C, Williams H, Gross JJ (2012) See what you think: reappraisal modulates behavioral and neural responses to social stimuli. Psychol Sci 23(4): 346e353. 10.1177/0956797612438559 10.1177/0956797612438559 22431908

[CR14] Buck B, Ludwig K, Meyer PS, Penn DL (2014) The use of narrative sampling in the assessment of social cognition: the narrative of emotions task (NET). Psychiatry Res 217(3):233–239 24726270 10.1016/j.psychres.2014.03.014 PMC4041701

[CR15] Caharel S, Bernard C, Thibaut F, Haouzir S, Di Maggio-Clozel C, Allio G, Fouldrin G, Petit M, Lalonde R, Rebaï M (2007) The effects of familiarity and emotional expression on face processing examined by ERPs in patients with schizophrenia. Schizophr Res 95:186–196 17644314 10.1016/j.schres.2007.06.015

[CR16] Carretié L, Kessel D, Carboni A, López-Martín S, Albert J, Tapia M, Hinojosa JA (2013) Exogenous attention to facial vs non-facial emotional visual stimuli. Soc Cogn Affect 8(7):764–773. 10.1093/scan/nss068 10.1093/scan/nss068 PMC3791067 22689218

[CR17] Carver CS, Harmon-Jones E (2009) Anger is an approach-related affect: evidence and implications. Psychol Bull 135:183–204. 10.1037/a0013965 19254075 10.1037/a0013965

[CR18] Carver R (1983) “A small, good thing”. In: Cathedral, New York: Knopf, pp. 59–89 (trad. it. Cattedrale, Duranti R, Torino: Einaudi editore, 2014)

[CR19] Cecchini M, Aceto P, Altavilla D, Palumbo L, Lai C (2013) The role of the eyes in processing an intact face and its scrambled image: a dense array ERP and low-resolution electromagnetic tomography (sLORETA) study. Soc Neurosci 8(4):314–325. 10.1080/17470919.2013.797020 23706064 10.1080/17470919.2013.797020

[CR20] Chiera A, Adornetti I, Altavilla D, Acciai A, Cosentino E, Deriu V, Ferretti F (2022) Does the character-based dimension of stories impact narrative processing? An event-related potentials (ERPs) study. Cogn Process 23(2):255–267. 10.1007/s10339-021-01070-1 35048215 10.1007/s10339-021-01070-1

[CR21] Choi D, Watanuki S (2014) Effect of empathy trait on attention to faces: an event-related potential (ERP) study. J Physiol Anthropol 33:1–8. 10.1186/1880-6805-33-4 24460950 10.1186/1880-6805-33-4 PMC3904693

[CR22] Damasio AR (1994) Descartes’ error. Grosset/Putnam

[CR23] Davis MH (1980) Interpersonal reactivity index

[CR24] De Jaegher H, Di Paolo E, Gallagher S (2010) Can social interaction constitute social cognition? Trends Cogn Sci 14(10):441–447. 10.1016/j.tics.2010.06.009 20674467 10.1016/j.tics.2010.06.009

[CR25] Demaree HA, Everhart DE, Youngstrom EA, Harrison DW (2005) Brain lateralization of emotional processing: historical roots and a future incorporating “dominance.” Behav Cogn Neurosci Rev 4:3–20. 10.1177/1534582305276837 15886400 10.1177/1534582305276837

[CR26] Dennis TA, Chen CC (2007) Neurophysiological mechanisms in the emotional modulation of attention: the interplay between threat sensitivity and attentional control. Biol Psychol 76(1–2):1–10 17582673 10.1016/j.biopsycho.2007.05.001 PMC2745961

[CR27] Djikic M, Oatley K, Moldoveanu MC (2013) Reading other minds: effects of literature on Empathy. Sci Study Lit 3(1):28–14. 10.1075/ssol.3.1.06dji

[CR28] Dvash J, Shamay-Tsoory SG (2014) Theory of mind and empathy as multidimensional constructs: neurological foundations. Top Lang Disord 34(4):282–295

[CR29] Eekhof LS, Van Krieken K, Willems RM (2022) Reading about minds: the social-cognitive potential of narratives. Psychon Bull Rev 29(5):1703–1718 35318585 10.3758/s13423-022-02079-z PMC9568452

[CR30] Earls HA, Curran T, Mittal V (2016) Deficits in early stages of face processing in schizophrenia: a systematic review of the P100 component. Schizophr Bull 42(2):519–527 26175474 10.1093/schbul/sbv096 PMC4753590

[CR31] Ernst LH, Ehlis AC, Dresler T, Tupak SV, Weidner A, Fallgatter AJ (2013) N1 and N2 ERPs reflect the regulation of automatic approach tendencies to positive stimuli. Neurosci Res 75(3):239–249. 10.1016/j.neures.2012.12.005 23298530 10.1016/j.neures.2012.12.005

[CR32] Ferstl EC, von Cramon DY (2002) What does the frontomedian cortex contribute to language processing: coherence or theory of mind? NeuroImage 17:1599–612. 10.1006/nimg.2002.1247 12414298 10.1006/nimg.2002.1247

[CR118] Ferretti F (2022) Narrative persuasion. Springer, Cham

[CR33] Foroni F, Mayr U (2005) The power of a story: New, automatic associations from a single reading of a short scenario. Psychon Bull Rev 12(1):139–144. 10.3758/BF03196359 15948288 10.3758/bf03196359

[CR34] Foti D, Olvet DM, Klein DN, Hajcak G (2010) Reduced electrocortical response to threatening faces in major depressive disorder. Depress Anxiety 27(9): 813e820. 10.1002/da.20712 10.1002/da.20712 20577985

[CR35] Frith CD, Frith U (2008) Implicit and explicit processes in social cognition. Neuron 60(3):503–510. 10.1016/j.neuron.2008.10.032 18995826 10.1016/j.neuron.2008.10.032

[CR36] Gadea M, Espert R, Salvador A, Marti-Bonmati L (2011) The sad, the angry, and the asymmetrical brain: dichotic listening studies of negative affect and depression. Brain Cogn 76:294–299. 10.1016/j.bandc.2011.03.003 21482001 10.1016/j.bandc.2011.03.003

[CR37] Green MC, Brock TC (2013) Transport narrative questionnaire. Measurement instrument database for the social science. J Personal Soc Psychol 79

[CR38] Green MC, Brock TC (2000) The role of transportation in the persuasiveness of public narratives. J Pers Soc Psychol 79:701. 10.1037/0022-3514.79.5.701 11079236 10.1037//0022-3514.79.5.701

[CR39] Hakemulder J (2000) The moral laboratory: experiments examining the effects of reading literature on social perception and moral self-concept (vol. 34). John Benjamins

[CR40] Happé F, Cook JL, Bird G (2017) The structure of social cognition: In (ter) dependence of sociocognitive processes. Annu Rev Psychol 68:243–267. 10.1146/annurev-psych-010416-044046 27687121 10.1146/annurev-psych-010416-044046

[CR41] Hemingway E (1925)"Big two-hearted river” Parts 1–2, in In Our Time, E. Hemingway, New York: Boni and Liveright edition

[CR42] Herbert C, Sfärlea A, Blumenthal T (2013) Your emotion or mine: labeling feelings alters emotional face perception—an ERP study on automatic and intentional affect labeling. Front hum Neurosci 7:378. 10.3389/fnhum.2013.00378 23888134 10.3389/fnhum.2013.00378 PMC3719026

[CR43] Herman D (2013) Storytelling and the sciences of mind. MIT Press, Cambridge

[CR44] Hillyard SA, Mangun GR, Woldorff MG, Luck SJ (1995) Neural mechanisms mediating selective attention. In: Gazzaniga MS (ed) The Cognitive Neurosciences. MIT Press, Cambridge, pp 320–67

[CR45] Hillyard SA, Vogel EK, Luck SJ (1998) Sensory gain control (amplification) as a mechanism of selective attention: electro-physiological and neuroimaging evidence. Philosoph Trans R Soc Lond B Biol Sci 353(1373):1257–270. 10.1098/rstb.1998.0281 10.1098/rstb.1998.0281 PMC1692341 9770220

[CR46] Hinojosa JA, Mercado F, Carretié L (2015) N170 sensitivity to facial expression: a meta-analysis. Neurosci Biobehav Rev 55:498–509 26067902 10.1016/j.neubiorev.2015.06.002

[CR47] Hodson G, Choma BL, Costello K (2009) Experiencing alien-nation: effects of a simulation intervention on attitudes toward homosexuals. J Exp Soc Psychol 45(4):974–978. 10.1016/j.jesp.2009.02.010

[CR48] Itier RJ, Neath-Tavares KN (2017) Effects of task demands on the early neural processing of fearful and happy facial expressions. Brain Res. 1663: 38–50. 10.1016/j.brainres.2017.03.013. Epub 2017 Mar 14. PMID: 28315309; PMCID: PMC5756067 10.1016/j.brainres.2017.03.013 PMC5756067 28315309

[CR50] Itier RJ, Taylor MJ (2004a) N170 or N1? Spatiotemporal differences between object and face processing using ERPs. Cereb Cortex 14:132–142. 10.1093/cercor/bhg111 14704210 10.1093/cercor/bhg111

[CR51] Itier RJ, Taylor MJ (2004b) Source analysis of the N170 to faces and objects. Neuroreport 15:1261–1265. 10.1097/01.wnr.0000127827.73576.d8 15167545 10.1097/01.wnr.0000127827.73576.d8

[CR52] Jack RE, Schyns PG (2015) The human face as a dynamic tool for social communication. Curr Biol 25(14):R621–R634. 10.1016/j.cub.2015.05.052 26196493 10.1016/j.cub.2015.05.052

[CR53] Johnson DR (2012) Transportation into a story increases empathy, prosocial behavior, and perceptual bias towards fearful expressions. Pers Individ Dif 52:150–155. 10.1016/j.paid.2011.10.005

[CR54] Kanade T, Cohn JF, Tian Y (2000) Comprehensive database for facial expression analysis. Proceedings of the Fourth IEEE International Conference on Automatic Face and Gesture Recognition (FG'00), Grenoble, France, 46–53

[CR55] Karl C, Hewig J, Osinsky R (2016) Passing faces: sequence dependent variations in the perceptual processing of emotional faces. Soc Neurosci 11(5): 531e544. 10.1080/17470919.2015.1115776 10.1080/17470919.2015.1115776 26599470

[CR56] Kidd DC, Castano E (2013) Reading literary fiction improves theory of mind. Science 342:377–379. 10.1126/science.1239918 24091705 10.1126/science.1239918

[CR57] Kolassa IT, Miltner WH (2006) Psychophysiological correlates of face processing in social phobia. Brain Res 1118:130–141 16970928 10.1016/j.brainres.2006.08.019

[CR58] Koopman EM (2015) Empathic reactions after reading: The role of genre, personal factors and affective responses. Poetics 50:62–79. 10.1016/j.poetic.2015.02.008

[CR59] Kuperberg GR, Lakshmanan BM, Caplan DN, Holcomb PJ (2006) Making sense of discourse: an fMRI study of causal inferencing across sentences. Neuroimage 33(1):343–361. 10.1016/j.neuroimage.2006.06.001 16876436 10.1016/j.neuroimage.2006.06.001

[CR60] Lang PJ, Bradley MM, Cuthbert BN (1997) International affective picture system (IAPS): Technical manual and affective ratings. NIMH Center Study Emot Attent 1(39–58):3

[CR61] Liu T, Pinheiro A, Zhao Z, Nestor PG, McCarley RW, Niznikiewicz MA (2012) Emotional cues during simultaneous face and voice processing: electrophysiological insights. Plos One, 7(2), Article e31001. 10.1371/journal.pone.0031001 10.1371/journal.pone.0031001 PMC3285164 22383987

[CR62] Lucey P, Cohn JF, Kanade T, Saragih J, Ambadar Z, Matthews I (2010) The Extended Cohn-Kanade Dataset (CK+): a complete expression dataset for action unit and emotion-specified expression*.* Proceedings of the Third International Workshop on CVPR for Human Communicative Behavior Analysis (CVPR4HB 2010), San Francisco, USA, 94–101

[CR63] Luck SJ, Hillyard SA (1994) Electrophysiological correlates of feature analysis during visual search. Psychophysiology 31(3):291–308. 10.1111/j.1469-8986.1994.tb02218.x 8008793 10.1111/j.1469-8986.1994.tb02218.x

[CR64] Mar RA (2018) Stories and the promotion of social cognition. Curr Direct Psychol Sci 27(4):257–262

[CR65] Mar RA (2018) Evaluating whether stories can promote social cognition: introducing the social processes and content entrained by narrative (SPaCEN) framework. Discourse Processes 55(5–6):454–479. 10.1080/0163853X.2018.1448209

[CR66] Mar RA, Oatley K, Djikic M, Mullin J (2010) Emotion and narrative fiction: interactive influences before, during, and after reading. Cogn Emot 25:818–833. 10.1080/02699931.2010.515151 10.1080/02699931.2010.515151 21824023

[CR67] Mar RA, Oatley K, Hirsh J, Dela Paz J, Peterson JB (2006) Bookworms versus nerds: exposure to fiction versus non-fiction, divergent associations with social ability, and the simulation of fictional social worlds. J Res Pers 40:694–712

[CR68] Mar RA, Oatley K, Peterson JB (2009) Exploring the link between reading fiction and empathy: ruling out individual differences and examining outcomes. Communications 34:407–428. 10.1515/COMM.2009.025

[CR69] Mar K, Oatley K (2008) The function of fiction is the abstraction and simulation of social experience. Perspect Psychol Sci 3(3):173–192. 10.1111/j.1745-6924.2008.00073 26158934 10.1111/j.1745-6924.2008.00073.x

[CR70] Mar RA, Oatley K, Djikic M, Mullin J (2011) Emotion and narrative fiction: Interactive influences before, during, and after reading. Cogn Emot 25(5):818–833. 10.1080/02699931.2010.515151 21824023 10.1080/02699931.2010.515151

[CR71] Mason RA, Just MA (2006) Neuroimaging contributions to the understanding of discourse processes. In: Traxler JM, Gernsbacher MA (eds) Handbook of psycholinguistics (2nd ed.). Academic Press, 765–799. 10.1016/B978-012369374-7/50020-1

[CR72] Mason RA, Just MA (2009) The role of the theory-of-mind cortical network in the comprehension of narratives. Lang Linguist Compass 3(1):157–174. 10.1111/j.1749-818X.2008.00122.x 19809575 10.1111/j.1749-818X.2008.00122.x PMC2756681

[CR73] Mason RA, Williams DL, Kana RK, Minshew N, Just MA (2008) Theory of mind disruption and recruitment of the right hemisphere during narrative comprehension in autism. Neuropsychologia 46(1):69–80. 10.1016/j.neuropsychologia.2007.07.018 10.1016/j.neuropsychologia.2007.07.018 PMC2259382 17869314

[CR74] Massaro G, Altavilla D, Aceto P, Pellicano GR, Lucarelli G, Luciani M, Lai C (2018) Neurophysiological correlates of collective trauma recall in 2009 L’Aquila earthquake survivors. J Trauma Stress 31(5):687–697. 10.1002/jts.22334 30338570 10.1002/jts.22334

[CR75] Meaux E, Roux S, Batty M (2014) Early visual ERPs are influenced by individual emotional skills. Soc Cogn Affect 9(8):1089–1098. 10.1093/scan/nst084 10.1093/scan/nst084 PMC4127009 23720573

[CR76] McPartland J, Cheung CH, Perszyk D, Mayes LC (2010) Face-related ERPs are modulated by point of gaze. Neuropsychologia 48(12):3657–3660. 10.1016/j.neuropsychologia.2010.07.020 20654637 10.1016/j.neuropsychologia.2010.07.020 PMC2949471

[CR77] Montag C, Panksepp J (2016) Primal emotional-affective expressive foundations of human facial expression. Motiv Emot 40:760–766. 10.1007/s11031-016-9570-x

[CR78] Montag C, Panksepp J (2017) Primary emotional systems and personality: an evolutionary perspective. Front psychol 8:464. 10.3389/fpsyg.2017.00464 28443039 10.3389/fpsyg.2017.00464 PMC5387097

[CR79] Moradi A, Mehrinejad SA, Ghadiri M, Rezaei F (2017) Event-related potentials of bottom-up and top-down processing of emotional faces. Basic Clin Neurosci 8(1):27 28446947 10.15412/J.BCN.03080104 PMC5396170

[CR80] Moratti S, Saugar C, Strange BA (2011) Prefrontal-occipitoparietal coupling underlies late latency human neuronal responses to emotion. J Neurosci 31(47):17278–86. 10.1523/JNEUROSCI.2917-11.2011.PMID:22114294;PMCID:PMC6623838 22114294 10.1523/JNEUROSCI.2917-11.2011 PMC6623838

[CR81] Müller-Bardorff M, Bruchmann M, Mothes-Lasch M, Zwitserlood P, Schlossmacher I, Hofmann D, Straube T (2018) Early brain responses to affective faces: a simultaneous EEG-fMRI study. NeuroImage 178:660–667. 10.1016/j.neuroimage.2018.05.081 29864521 10.1016/j.neuroimage.2018.05.081

[CR82] Oatley K (1995) A taxonomy of the emotions of literary response and a theory of identification in fictional narrative. Poetics 23(1–2):53–74. 10.1016/0304-422X(94)P4296-S

[CR83] Oatley K (1999) Why fiction may be twice as true as fact: fiction as cognitive and emotional simulation. Rev Gen Psychol 3:101–117. 10.1037/1089-2680.3.2.101

[CR84] Oruc I, Balas B, Landy MS (2019) Face perception: a brief journey through recent discoveries and current directions. Vis Res 157:1–9. 10.1016/j.visres.2019.06.005 31201832 10.1016/j.visres.2019.06.005 PMC7371014

[CR85] Oruc I, Balas B, Landy MS (2019) Introduction to the special issue on face perception: experience, models, and neural mechanisms. Vis Res 157:10–11. 10.1016/j.visres.2019.06.001 31173774 10.1016/j.visres.2019.06.001

[CR86] Palomero-Gallagher N, Amunts K (2022) A short review on emotion processing: a lateralized network of neuronal networks. Brain Struct Funct 227(2):673–684. 10.1007/s00429-021-02331-7 34216271 10.1007/s00429-021-02331-7 PMC8844151

[CR87] Paluck EL (2009) Reducing intergroup prejudice and conflict using the media: a field experiment in Rwanda. J Pers Soc Psychol 96:574–587. 10.1037/a0011989 19254104 10.1037/a0011989

[CR88] Panksepp J (1998) Affective neuroscience: The foundations of human and animal emotions. Oxford University Press, New York

[CR89] Patin A, Hurlemann R (2015) Social cognition. Cogn Enhanc. 10.1007/978-3-319-16522-6_10

[CR90] Petroni A, Canales-Johnson A, Urquina H, Guex R, Hurtado E, Blenkmann A, Ibanez A (2011) The cortical processing of facial emotional expression is associated with social cognition skills and executive functioning: a preliminary study. Neurosci Lett 505(1):41–46. 10.1016/j.neulet.2011.09.062 22001365 10.1016/j.neulet.2011.09.062

[CR91] Picton TW, Bentin S, Berg P, Donchin E, Hillyard SA, Johnson R, Miller GA, Ritter W, Ruchkin DS, Rugg MD, Taylor MJ (2000) Guidelines for using human event-related potentials to study cognition: recording standards and publication criteria. Psychophysiol 37(2):127–152. 10.1111/1469-8986.3720127 10731765

[CR92] Pinkham AE, Penn DL, Green MF, Buck B, Healey K, Harvey PD (2014) The social cognition psychometric evaluation study: results of the expert survey and RAND panel. Schizophr Bull 40(4):813–23. 10.1093/schbul/sbt081 23728248 10.1093/schbul/sbt081 PMC4059426

[CR93] Pourtois G, Grandjean D, Sander D, Vuilleumier P (2004) Electrophysiological correlates of rapid spatial orienting towards fearful faces. Cereb Cortex 14:619–633 15054077 10.1093/cercor/bhh023

[CR94] Rellecke J, Sommer W, Schacht A (2012) Does processing of emotional facial expressions depend on intention? Time-resolved evidence from event-related brain potentials. Biol Psychol 90(1):23–32. 10.1016/j.biopsycho.2012.02.002 22361274 10.1016/j.biopsycho.2012.02.002

[CR95] Roussel GA, Macé MJ, Fabre-Thorpe M (2004) Spatiotemporal analyses of the N170 for human faces, animal faces and objects in natural scenes. Neuroreport 15:2607–2611 15570161 10.1097/00001756-200412030-00009

[CR96] Sadeh B, Podlipsky I, Zhdanov A, Yovel G (2010) Event-related potential and functional MRI measures of face-selectivity are highly correlated: a simultaneous ERP-fMRI investigation. Hum Brain Mapp 31:1490–1501. 10.1002/hbm.20952 20127870 10.1002/hbm.20952 PMC6870976

[CR97] Santos IM, Iglesias J, Olivares EI, Young AW (2008) Differential effects of object-based attention on evoked potentials to fearful and disgusted faces. Neuropsychologia 46(5):1468–1479. 10.1016/j.neuropsychologia.2007.12.024 18295286 10.1016/j.neuropsychologia.2007.12.024

[CR98] Schindler S, Bublatzky F (2020) Attention and emotion: an integrative review of emotional face processing as a function of attention. Cortex 130:362–386. 10.1016/j.cortex.2020.06.010 32745728 10.1016/j.cortex.2020.06.010

[CR99] Schindler S, Bruchmann M, Straube T (2023) Beyond facial expressions: a systematic review on effects of emotional relevance of faces on the N170. Neurosci Biobehav Rev 153:105399 37734698 10.1016/j.neubiorev.2023.105399

[CR100] Schore AN (2005) A neuropsychoanalytic viewpoint: Commentary on paper by Steven H. Knoblauch. Psychoanal Dialog 15(6):829–854. 10.2513/s10481885pd1506_3

[CR101] Sestir M, Green M (2010) You are who you watch: identification and transportation effects on temporary self-concept. Soc Influ 5(4):272–288. 10.1080/15534510.2010.490672

[CR102] Shah D, Knott V, Baddeley A, Bowers H, Wright N, Labelle A, Collin C (2018) Impairments of emotional face processing in schizophrenia patients: evidence from P100, N170 and P300 ERP components in a sample of auditory hallucinators. Int J Psychophysiol 134:120–134 30291891 10.1016/j.ijpsycho.2018.10.001

[CR103] Smith E, Weinberg A, Moran T, Hajcak G (2013) Electrocortical responses to NIMSTIM facial expressions of emotion. Int J Psychophysiol 88(1):17–25. 10.1016/j.ijpsycho.2012.12.004 23280304 10.1016/j.ijpsycho.2012.12.004

[CR104] Sturani M (2013) Pietre, piume e insetti. L’arte di raccontare la natura, Torino: Einaudi editore

[CR105] Syrjänen E, Wiens S, Fischer H, Zakrzewska M, Wartel A, Larsson M, Olofsson JK (2018) Background odors modulate N170 ERP component and perception of emotional facial stimuli. Front psychol 9:322954. 10.3389/fpsyg.2018.01000 10.3389/fpsyg.2018.01000 PMC6029154 29997539

[CR106] Tortosa MI, Lupianez J, Ruz M (2013) Race, emotion and trust: an ERP study. Brain Res 1494:44–55. 10.1016/j.brainres.2012.11.037 23220554 10.1016/j.brainres.2012.11.037

[CR107] Van Krieken K, Hoeken H, Sanders J (2017) Evoking and measuring identification with narrative characters: a linguistic cues framework. Front Psychol 8:1190. 10.3389/fpsyg.2017.01190 28751875 10.3389/fpsyg.2017.01190 PMC5507957

[CR108] Van Laer T, De Ruyter K, Visconti LM, Wetzels M (2013) The extended transportation-imagery model: a meta-analysis of the antecedents and consequences of consumers’ narrative transportation. J Consum Res 40:797–817. 10.1086/673383

[CR109] Virtue S, Haberman J, Clancy Z, Parrish T, Jung-Beeman M (2006) Neural activity of inferences during story comprehension. Brain Res 1084(1):104–14. 10.1016/j.brainres.2006.02.053 16574079 10.1016/j.brainres.2006.02.053

[CR110] Walkington Z, Wigman SA, Bowles D (2020) The impact of narratives and transportation on empathic responding. Poetics 80:101425

[CR111] Walter H (2012) Social cognitive neuroscience of empathy: Concepts, circuits, and genes. Emot Rev 4(1):9–17

[CR112] Westra E, Carruthers P (2018) Theory of mind. In: Shackelford TH, Weekes-Shackelford VA (eds), Encyclopedia of Evolutionary Psychological Science, Springer, 1–7. 10.1007/978-3-319-16999-6_2376-1

[CR113] Wieser MJ, Brosch T (2012) Faces in context: a review and systematization of contextual influences on affective face processing. Front Psychol. 10.3389/fpsyg.2012.00471 23130011 10.3389/fpsyg.2012.00471 PMC3487423

[CR114] Williams LM, Liddell BJ, Rathjen J, Brown KJ, Gray J, Phillips M, Young A, Gordon E (2004) Mapping the time course of nonconscious and conscious perception of fear: an integration of central and peripheral measures. Hum Brain Mapp 21:64–74 14755594 10.1002/hbm.10154 PMC6871876

[CR115] Wronka E, Walentowska W (2011) Attention modulates emotional expression processing. Psychophysiology 48(8):1047–1056. 10.1111/j.1469-8986.2011.01180.x 21332489 10.1111/j.1469-8986.2011.01180.x

[CR116] Wu L, Muller HJ, Zhou X, Wei P (2019) Differential modulations of reward expectation on implicit facial emotion processing: ERP evidence. Psychophysiol 56(3):e13304. 10.1111/psyp.13304 10.1111/psyp.13304 30443953

[CR117] Zhang D, Wang L, Luo Y, Luo Y (2012) Individual differences in detecting rapidly presented fearful faces. Plos One 7(11):e49517. 10.1371/journal.pone.004 23166693 10.1371/journal.pone.0049517 PMC3498139

